# Negative Coupling as a Mechanism for Signal Propagation between C2 Domains of Synaptotagmin I

**DOI:** 10.1371/journal.pone.0046748

**Published:** 2012-10-05

**Authors:** Michael E. Fealey, Jacob W. Gauer, Sarah C. Kempka, Katie Miller, Kamakshi Nayak, R. Bryan Sutton, Anne Hinderliter

**Affiliations:** 1 Department of Chemistry and Biochemistry, University of Minnesota Duluth, Duluth, Minnesota, United States of America; 2 Department of Cell Physiology, Texas Tech University Health Sciences Center, Lubbock, Texas, United States of America; University of South Florida College of Medicine, United States of America

## Abstract

Synaptotagmin I (Syt I) is a vesicle-localized protein implicated in sensing the calcium influx that triggers fast synchronous release of neurotransmitter. How Syt I utilizes its two C2 domains to integrate signals and mediate neurotransmission has continued to be a controversial area of research, though prevalent hypotheses favor independent function. Using differential scanning calorimetry and fluorescence lifetime spectroscopy in a thermodynamic denaturation approach, we tested an alternative hypothesis in which both domains interact to cooperatively disseminate binding information. The free energy of stability was determined for C2A, C2B, and C2AB constructs by globally fitting both methods to a two-state model of unfolding. By comparing the additive free energies of C2A and C2B with C2AB, we identified a negative coupling interaction between the C2 domains of Syt I. This interaction not only provides a mechanistic means for propagating signals, but also a possible means for coordinating the molecular events of neurotransmission.

## Introduction

Regulated exocytosis of neurotransmitter requires the fusion of synaptic vesicles with the plasma membrane of the presynaptic neuron. This complex process is mediated by several key proteins including synaptobrevin, syntaxin-1, SNAP-25, complexins, and synaptotagmin I [1]–[5]. Synaptotagmin I (Syt I), a vesicle-localized protein, has been strongly implicated in sensing the calcium (Ca^2+^) influx that ultimately triggers vesicle and plasma membrane fusion [6]–[8]. Syt I consists of a short luminal N-terminus, a transmembrane region, and two cytosolic C2 domains in tandem known as C2A and C2B. Both domains bind Ca^2+^ and acidic phospholipids [9]–[12] like phosphatidylserine (PS) and phosphatidylinositol (PIP_2_), two lipids that modulate fusion [13]. In addition to binding Ca^2+^ and lipid, Syt I also interacts with several proteins involved in vesicle fusion, including members of the SNARE complex [14]–[17].

How Syt I utilizes two C2 domains to rapidly transmute binding information from Ca^2+^ and lipid ligands as well as from proteins within its immediate vicinity to facilitate vesicle and plasma membrane fusion is not well understood, with conflicting evidence both for and against domain cooperation [9], [15], [18]–[22]. It has been suggested, however, that the function of tandem lipid-binding domains may be one of coincidence detection [23]. In this framework, the differential binding preferences of each domain allow for appropriate temporal and spatial positioning of the protein and, in the case of Syt I, components needed for fusion. This mechanistic view as it applies to C2 domains implies that tethering C2A to C2B would result in their additive, independent function.

Recent theoretical work [24], [25] provides an alternative, cooperative function for two domain signaling proteins. Briefly, to both detect a signal (which relies on redistributing domain conformations or conformers [26]) and propagate it, the protein needs to have a unique set of domain stabilities and a free energy of interaction (Δg_int_) that collectively facilitate coupling. The sign of Δg_int_ describes the nature of the inter-domain coupling and how the domains communicate with one another. In positive coupling, both domains will experience a similar effect upon introducing a perturbation. With negative coupling, the two domains experience opposite effects. If, for instance, the perturbation is ligand binding and Δg_int_ is positive, both domains will experience a stabilizing effect. Regardless of Δg_int_ sign, introducing a perturbation that changes the conformer distribution (also known as an ensemble) of one domain will directly impact the ensemble of the adjacent domain.

In light of the theoretical work above, we propose that rather than having an additive coincidence detector function, the C2 domains of Syt I cooperatively propagate binding information through inter-domain coupling. This type of interaction would be of significant functional relevance as each domain would become an allosteric regulator of the other. Ligand binding to C2B, for instance, would not only redistribute C2B’s ensemble, but would also increase or decrease the probability of binding-competent conformers of C2A being occupied.

**Figure 1:**
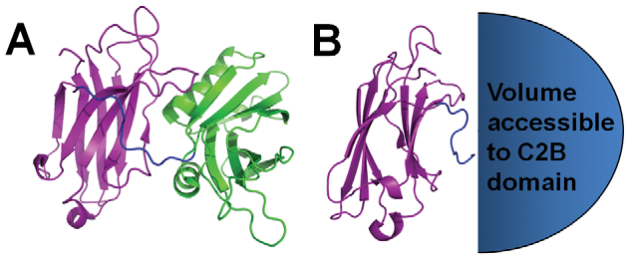
Restricted volume effect of domain tethering. (A) Crystal structure of Syt I in the absence of ligand (PDB 2R83). The C2A domain, nine-residue linker region, and C2B domain are colored purple, blue, and green, respectively. (B) Conceptual representation and illustrative calculation of the volume accessible to the C2B domain with respect to C2A. Because the C2B domain is tethered to C2A, the volume it can occupy is restricted. This significantly increases the local concentration of C2B. If, for instance, the volume is calculated using the length of the linker and width of C2B as an approximate hemisphere radius (Pymol measurement of roughly 57 Å), the accessible volume is 4×10^−19^ cm^3^. Assuming 1 molecule of C2B occupies this volume, its local concentration is equal to (1 molecule C2B/6.022×10^23^ molecules per mole)/(4×10^−25^ m^3^) or ∼4 M. The local concentration of the C2A domain can be approximated in an analogous way. The resulting concentrations would shift the association equilibrium in the direction of bound domains.

Testing for the proposed interaction and quantifying its energetic value is difficult because of Δg_int_’s necessarily small magnitude. This problem is best appreciated in the context of the C2 domain’s restricted volume. The nine residue linker that tethers C2A and C2B together (Fig. 1A) significantly reduces accessible solution volume, causing the local concentration of each domain to increase well beyond attainable solution concentrations (Fig. 1B). Consequently, there is an increased probability of domain association. If Δg_int_ were large relative to the two domains in this context, then C2A and C2B would predominantly be in an associated state; this arrangement could greatly reduce the conformational freedom of the individual domains and negatively impact the signaling capacity of the protein. To preserve this function, Δg_int_ would need to be small.

Since our hypothesis is based upon subtle differences in protein energetics, two highly sensitive methods were employed to test it, namely differential scanning calorimetry (DSC) [27], [28] and fluorescence lifetime spectroscopy (FLT) [26], the latter of which selectively monitors tryptophan residue fluorescence. Constructs of C2A, C2B, and C2AB (containing just the cytosolic C2 domains) were thermally denatured using each technique. The resulting DSC and FLT data sets were analyzed by globally fitting both to a two-state model of protein unfolding (see Text S1). The fit parameters of enthalpy (ΔH_Tm_), change in heat capacity (ΔC_p_), and melting temperature (T_m_) were then used to determine free energies of stability at physiological temperature (ΔG°_37°C_) using the Gibbs-Helmholtz equation (equation (4)). The validity of the two-state model was judged by comparison of experimental (ΔH_cal_) and calculated (ΔH_Tm_) enthalpies. By comparing the additive free energies of the individual C2 domains with the C2AB construct, we were able to identify a negative coupling interaction between the C2 domains of Syt I. The quantitative description of this interaction provides a mechanistic means by which Syt I cooperatively propagates binding information throughout both domains and may represent a capacity for coordinating the molecular events of neurotransmitter release.

## Results

### Separate C2 Domains are Weak and Conformationally Flexible

**Figure 2:**
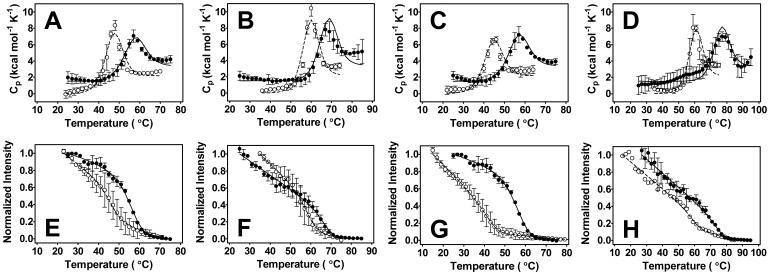
Globally-fit denaturation of C2A (•) and C2B (○) using DSC (top) and FLT (bottom) methods. Circles represent the raw data and lines are the fitted model. C2A denaturations displayed with permission from Biophysical Journal. Higher concentrations refer to DSC samples. (A, E) [C2A] = 13 µM and 0.75 µM, [C2B] = 13 µM and 0.80 µM in the presence of 500 µM EGTA. (B, F) [C2A] = 13 µM and 0.75 µM, [C2B] = 12 µM and 0.75 µM in the presence of Ca^2+^. [Ca^2+^] = 800 µM and 770 µM for C2A, [Ca^2+^] = 5.3 mM and 5.2 mM for C2B. (C, G) [C2A] = 13 µM and 0.75 µM, [C2B] = 15.0 µM and 3.2 µM in the presence of LUVs consisting of 60∶40 POPC:POPS. [Lipid] = 870 µM and 50 µM for C2A, [Lipid] = 1.1 mM and 240 µM for C2B. Higher [C2B] in FLT was needed under lipid conditions to better see transition. (D, H) [C2A] = 13 µM and 0.75 µM, [C2B] = 13 µM and 3.2 µM for C2B in the presence of both LUVs and Ca^2+^. For C2A, [Ca^2+^] = 800 µM and [lipid] = 870 µM, [Ca^2+^] = 770 µM and [lipid] = 50 µM. For C2B, [Ca^2+^] = 5.2 mM and [lipid] = 290 µM, [Ca^2+^] = 5.2 mM and [lipid] = 70 µM. Low [lipid] for C2B prevented precipitation in calorimeter, but also limited FLT data collection due to flocculation and light scattering [29].

To determine the relative stability of one domain compared to the other and whether or not the C2 domains of Syt I interact, the thermodynamic parameters of C2A and C2B in isolation were extracted from each domain’s thermal denaturation. The enthalpies measured for both C2A and C2B constructs did not show strong dependence on either concentration or scan rate consistent with the system reflecting equilibrium conditions (Tables 1 and 2, Table S1) [26], [29]. When comparing the denaturation profiles of C2A and C2B (Fig. 2) in the presence and absence of Ca^2+^ and lipid ligands, there are clear differences in domain stability, with C2B being generally less stable (Table 3). Note that relative to proteins of similar size, C2A and C2B have markedly low intrinsic stability [30], [31]. The two domains also differ from one another in extent of reversible folding, with C2B being minimally reversible (5–30%) as assessed by FLT (no discernible reversibility was found on any second denaturation scan of a DSC sample) as compared to C2A which exhibits nearly complete reversibility (approximately 60–90% by both FLT and DSC [26]).

**Table 1:** Concentration dependence controls for C2B and C2AB.

C2B (Scan Rate, 1°C/min)		C2AB (Scan Rate, 1°C/min)	
*Concentration (mM)*	ΔH_cal_ (kcal/mole)*	*Concentration (mM)*	ΔH_cal_ (kcal/mole)*
0.012	50.1	0.011	97.1
0.013	46.1	0.012	87.7
0.014	43.1	0.013	92.4
0.015	45.7	0.015	89.0
0.020	42.1	0.019	85.9
0.021	43.7	0.020	85.6
*Average ΔH_cal_ (kcal/mole)*	*Standard Deviation*	*Average ΔH_cal_ (kcal/mole)*	*Standard Deviation*
45.1	2.9	89.6	4.4
*ΔH_cal_ Range (kcal/mole)*		*ΔH_cal_ Range (kcal/mole)*	
37.5–53.2		85.2–104.7	

Footnote for Table 1: Note that * indicates enthalpies listed above came from averaging multiple denaturations at each specified concentration. For completeness, the overall range of enthalpies represented within the averaged values is also listed. All measured calorimetric enthalpies for both constructs can, however, be seen in Table S1 and Table S2.

**Table 2:** Scan rate dependence controls for C2B and C2AB.

C2B (Concentration 15 µM)		C2AB (Concentration 15 µM)	
*Scan Rate (°C/min)*	*ΔH_cal_ (kcal/mole)*	*Scan Rate (°C/min)*	*ΔH_cal_ (kcal/mole)*
1.00	42.1	1.00	89.0
1.10	45.7	1.10	86.4
1.15	52.5	1.15	90.8
1.20	49.9	1.20	87.0
*Average ΔH_cal_ (kcal/mole)*	*Standard Deviation*	*Average ΔH_cal_ (kcal/mole)*	*Standard Deviation*
47.5	4.6	88.3	2.0

Footnote for Table 2: In both cases, measured calorimetric enthalpies for both C2B and C2AB constructs did not strongly depend on scan rate. Analogous controls were performed for the C2A construct in a separate publication.

**Table 3:** Thermodynamic parameters and their associated error from the global fit of DSC and FLT data.

Protein (Environment)	ΔH_Tm_ (kcal/mole)	ΔG° at 37°C (kcal/mole)	ΔS (kcal/mole·K)	T_m_ (°C)	ΔH_Tm_/ΔH_cal_
*C2A (EGTA)*	58.7±0.3	2.32±0.05	0.18±0.01	56.0±0.1	0.98
*C2A (Ca^2+^)*	77.4±0.6	4.23±0.05	0.23±0.01	67.6±0.1	1.08
*C2A (PS)*	61±1	2.44±0.05	0.19±0.02	55.5±0.2	1.03
*C2A (Ca^2+^, PS)*	72.4±0.9	3.73±0.05	0.21±0.01	75.6±0.3	0.86
*C2B (EGTA)*	69.6±0.6	1.74±0.09	0.22±0.01	46.4±0.1	1.45
*C2B (Ca^2+^)*	81.8±0.5	3.78±0.10	0.25±0.01	59.0±0.1	1.39
*C2B (PS)*	63.7±0.1	1.13±0.02	0.20±0.01	43.3±0.1	1.51
*C2B (Ca^2+^, PS)*	75.4±0.2	3.41±0.01	0.23±0.01	59.4±0.1	1.92
*C2AB (EGTA)*	102.9±0.4	2.24±0.01	0.32±0.01	45.6±0.1	1.05
*C2AB (Ca^2+^)*	112.8±0.5	4.10±0.01	0.34±0.01	59.3±0.1	0.97
*C2AB (PS)*	86.8±0.4	1.74±0.01	0.27±0.01	45.1±0.1	1.05
C2AB (PIP_2_)*	198±2	4.09±0.45	0.62±0.01	44.2±0.1	NA
*C2AB (PIP_2_)^†^*	96.0±0.2	2.56±0.01	0.32±0.01	49.0±0.1	1.08
C2AB (Ca^2+^, PIP_2_)*	136±1	5.53±0.02	0.41±0.01	58.0±0.1	NA

Footnote for Table 3: Note that * indicates parameters obtained from fitting just FLT data sets and ^†^ indicates parameters obtained from fitting just DSC data sets. EGTA stands for ethylene glycol tetraacetic acid. NA stands for not applicable and is used for instances where just FLT data was fit. As an additional control, FLT signal was collected at 345 nm, normalized, and subsequently globally fit with DSC denaturation data. The thermodynamic parameters that resulted are similar to those presented above, indicating a lack of water fluorescence contribution at the 340 nm wavelength used for this analysis (Table S3).

**Figure 3:**
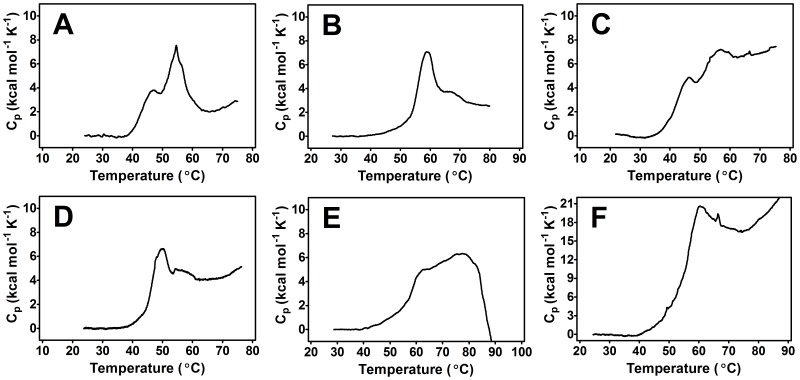
Raw DSC data for simultaneous thermal denaturation of separate C2A and C2B domains. (A) 10 µM of each domain in the presence of 500 µM EGTA. (B) 15 µM of each domain in the presence of 5.2 mM Ca^2+^ conditions. (C) 15 µM of each domain in the presence of 2.2 mM PS. (D) 11 µM of each domain in the presence of 2.9 mM PIP_2_. (E) 12 µM of each domain in the presence of 1.6 mM PS and 5.2 mM Ca^2+^. (F) 10 µM of each domain in the presence of 2.8 mM PIP_2_ and 5.2 mM Ca^2+^ conditions. Note that in most instances, two separate peaks are seen representing the independent unfolding of each domain. If C2A and C2B within the C2AB construct did not communicate, the C2AB denaturation profile would more closely resemble the above heat capacity profiles.

If C2A and C2B did not interact with one another when tethered together, the DSC denaturation profile would look similar to the superposition of both individual domain denaturations [25]. Such heat capacity profile controls were generated in both the presence and absence of Ca^2+^ and lipid ligands (Fig. 3). Despite extensive overlap of excess heat capacities, variable ΔC_p_, and in some instances terminal precipitation and large heat capacities, the two resultant peaks provide a reference denaturation profile to which the individual domains and the C2AB construct can be compared.

### Tethered C2 Domains are Weaker Still and Unfold as One

**Figure 4:**
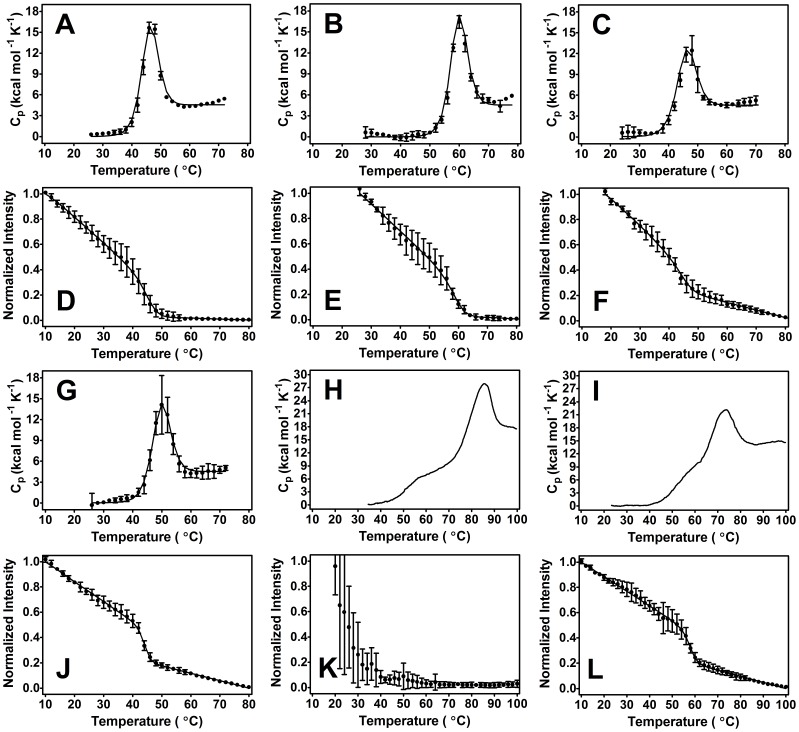
Thermal denaturation of C2AB using DSC (A–C, G–I) and FLT (D–F, J–L) methods. Circles represent raw data and lines are the fitted model, excluding panels (H) and (I) wherein the line represents raw heat capacity data. Large and small concentrations refer to DSC and FLT concentrations, respectively. (A, D) 13 µM and 4.5 µM C2AB in the presence of 500 µM EGTA. (B, E) 12 µM and 0.75 µM C2AB in the presence of 5.2 mM and 5.1 mM Ca^2+^. (C, F) 12 µM and 0.75 µM C2AB in the presence of 1.7 mM and 110 µM PS. (G, J) 11 µM (3 replicates of DSC) and 0.75 µM C2AB in the presence of 2.9 mM and 210 µM PIP_2_. (H, K) 12 µM C2AB in the presence of 5.2 mM Ca^2+^ and 1.7 mM PS; 0.75 µM C2AB in the presence of 5.1 mM Ca^2+^ and 110 µM PS. (F, I) 11 µM (1 replicate of DSC) C2AB in the presence of 5.2 mM Ca^2+^ and 2.9 mM PIP_2_; 0.75 µM C2AB in the presence of 5.1 mM Ca^2+^ and 210 µM PIP_2_. Both calorimetric denaturations involving PIP_2_ had a limited number of replicates due to precipitation. In the absence of any interaction, the two domains would unfold independently. Instead, here the two domains unfold as one.

**Figure 5:**
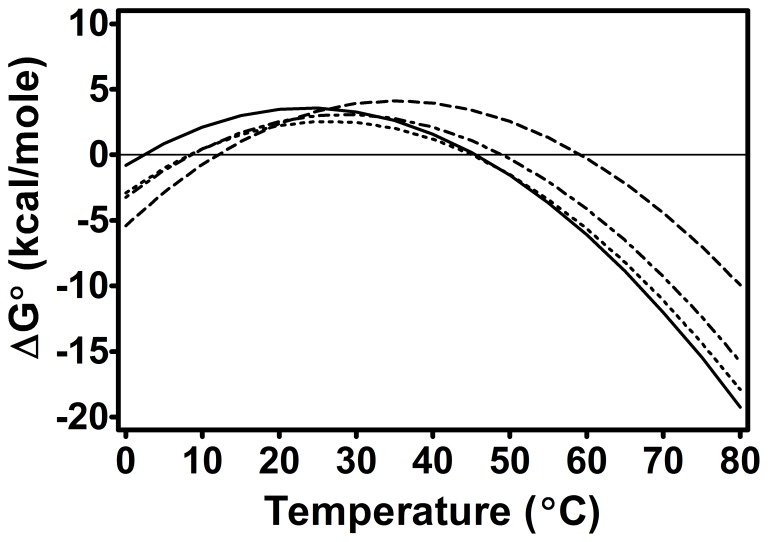
Stability of C2AB as a function of temperature in the presence and absence of ligand. Solid, dashed, dotted, and dash-dot-dash lines represent EGTA, Ca^2+^, phosphatidylserine, and phosphatidylinositol environments. Note that most proteins of comparable size have maxima in the range of 10–20 kcal/mole [30], [31].

The Syt I C2AB construct was thermally denatured in the presence and absence of Ca^2+^ and lipid ligands. The enthalpy measured for this construct, like the individual domains, did not show a strong dependence on concentration or scan rate (Tables 1 and 2, Table S2). In all environments, with the exception of combined Ca^2+^ and lipid ligands, the two domains of C2AB exhibit coupled unfolding (Fig. 4). This coupled unfolding is indicative of an inter-domain interaction and contrasts with the model heat capacity profiles of independent domain unfolding depicted in Figure 3. With regard to stability, note again that relative to other proteins of approximately the same size, C2AB is marginally stable (Table 3 and Fig. 5) [30], [31]. Annexin I, for instance, is another Ca^2+^ and membrane binding protein that has an estimated ΔG°_20°C_ of 11.5 kcal/mol (vs. 2 kcal/mol for C2AB at analogous temperature) [32]. Reversibility as assessed by FLT ranged from 1–24% (no discernible reversibility was seen in any second scan of a DSC sample).

### C2 Domains Interact through Inverse-Stabilization

When comparing the additive free energies of the isolated C2A and C2B domains with the C2AB construct, a Δg_int_ of −1.8±0.1 kcal/mol was found under ligand-free conditions at 37°C (Δg_int_ = ΔG°_C2AB_ – (ΔG°_C2A_ + ΔG°_C2B_)). Since Δg_int_<0, the C2 domains of Syt I exhibit a negative inter-domain coupling interaction and the two domains experience opposite stabilizing effects upon introducing a perturbation. This form of coupling is similar to that of other allosteric proteins recently reported [33], [34]. Furthermore, when repeating the same calculation for Ca^2+^ and PS conditions, Δg_int_ of −3.9±0.1 kcal/mole and −1.83±0.05 kcal/mole, respectively, were found. This indicates that the magnitude of negative coupling can change in response to ligand, a feature consistent with previous work showing that coupling can be modulated [35]. While the magnitude of Δg_int_ can change depending on the ligands present, it should be noted that the different combinations of Δg_int_, ΔG°_C2A_, and ΔG°_C2B_ within the tethered construct consistently facilitate domain coupling, though to varying extents.

### Syt I and its Domains are Malleable

The free energies of C2A, C2B, and C2AB in the presence of endogenous ligands were generally higher relative to a ligand-free environment (Table 3), with one exception. The binding of PS to C2B and C2AB had an overall destabilizing effect. C2AB, for instance, experienced a 0.5 kcal/mole decrease in free energy, nearly 23% of the ligand-free ΔG°_37°C_ value. This destabilization was more pronounced in C2B where the decrease in free energy was about 35% of the ligand-free ΔG°_37°C_ value. The phosphatidylinositol lipid PIP_2_, in contrast, stabilized C2AB.

The denaturation scans involving PIP_2_ differed between DSC and FLT methods (Fig. 4G, 4I, 4J, 4L). The C2AB FLT denaturations showed a sharp transition (occurring over a small temperature range) at a lower temperature in comparison to the analogous DSC denaturation. Fitting the two data sets separately generated ΔG°_37°C_ values of 4.09±0.45 and 2.56±0.01 kcal/mole for FLT and DSC, respectively. Additionally, in C2AB denaturations with PIP_2_ and Ca^2+^ ligands, DSC showed partial peak splitting whereas FLT showed one unfolding transition. The transition measured on FLT was sharp and when fit to a two-state model in isolation generated a ΔG°_37°C_ of 5.53±0.02 kcal/mole. PS also caused method discrepancy, though only in the presence of Ca^2+^ ligand. When C2AB was denatured using FLT in the presence of PS and Ca^2+^, no discernible transition could be seen (Fig. 4K).

## Discussion

**Figure 6:**
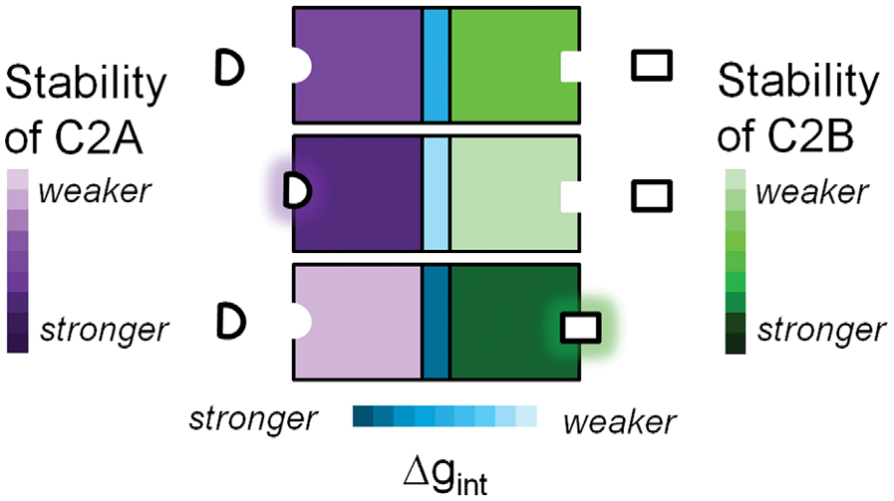
Conceptual representation of negative coupling. In the absence of bound ligand (top), domains have basal level stability. When a ligand specific to the C2A domain binds (middle), C2A is stabilized and C2B is destabilized through negative coupling. When a ligand specific to C2B binds (bottom), the opposite effect is seen; C2B is stabilized and C2A is destabilized. Note that binding of domain-specific ligands lowers the probability of binding-competent conformers being occupied in the adjacent domain through domain destabilization, representing a form of allosteric regulation. The extent of negative coupling, like domain stability, changes depending on the types of ligand present.

The purpose of the current study was to test a theory-driven hypothesis in which the C2 domains of Syt I interact to cooperatively disseminate binding information. In isolation, C2A and C2B were found to be energetically distinct. When tethered together, the two domains unfolded as one entity and were found to be less stable together than apart, indicative of a negative inter-domain coupling interaction. These results not only provide further experimental validation [33]–[37] of recent theoretical work [24], [25], but also a mechanistic means by which the C2 domains of Syt I propagate signal from endogenous ligands throughout both domains of the protein (Fig. 6) [38].

Aside from showing negative inter-domain coupling, the results presented here also indicate that the interaction is subject to modulation by Ca^2+^ and phospholipid ligands. The general observation of marginal stability in all three protein constructs relative to proteins of similar size indicates that Syt I has a larger degree of conformational freedom and thus is more flexible. Additionally, the isolated C2A and C2B domain free energies of stability (which report on the overall breadth of each domain’s conformational ensemble) in the presence and absence of various ligands indicates a high degree of malleability (Table 3). This is typified by the 1.8- and 0.6-fold changes in stability of C2A (in the presence of Ca^2+^ alone) and C2B (in the presence of PS alone), respectively. This malleability is enhanced when the two domains are tethered together and can be enhanced further still when a perturbation is introduced. For instance, the binding of Ca^2+^ ligand to the C2AB construct changes Δg_int_ from −1.8 kcal/mole to −3.9 kcal/mole. In other words, C2A and C2B are even less stable together than apart when in the presence of Ca^2+^. Qualitative application of the fluctuation dissipation theorem provides an additional representation for this malleability, showing ligand-induced changes in the relative positions of native and denatured enthalpy distributions (Text S1, Fig. S1) [39]. Overall, this degree of flexibility likely makes C2AB highly sensitive to other binding partners in its immediate environment, a trait conceptually consistent with a calcium sensor [26].

**Figure 7:**
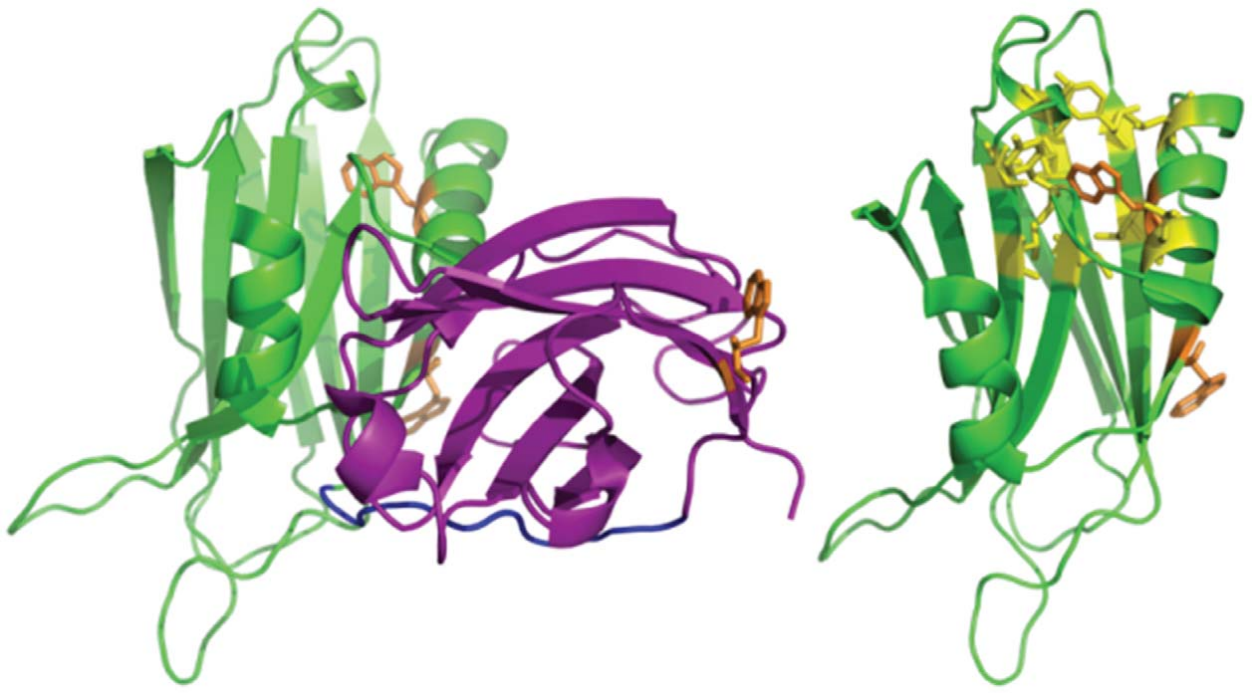
Location of tryptophan residues (orange) in C2A (purple) and C2B (green). Two of three tryptophan residues (C2A’s and one of C2B’s) occupy superficial positions and may, as a result, be more solvent exposed in solution (left). The second tryptophan in the C2B domain is partially embedded in the core of the β-sandwich motif amongst several hydrophobic residues (yellow) (right). The differences in tryptophan environment likely give rise to unequal FLT signal contributions, with most of the signal coming from C2B’s β-sandwich residue.

The differential binding preferences of each C2 domain can, however, modulate Δg_int_. In the presence of PS alone (which had an overall destabilizing effect, decreasing C2AB’s free energy from 2.24 kcal/mole to 1.74 kcal/mole), Δg_int_ did not change within error (went from −1.8 kcal/mole to −1.83 kcal/mole). Here, the binding of PS to C2A does not appear to have as great of a destabilizing effect on the adjacent C2B domain. If, however, Ca^2+^ ligand is added to drive the C2A domain into a lipid bound state, the degree of C2B destabilization becomes more pronounced. Evidence for this is seen in the FLT denaturations of C2AB under PS and Ca^2+^ conditions (Fig. 4K). Because two of C2AB’s three tryptophan residues are likely solvent exposed (Fig. 7) and the third residue is partially buried in the hydrophobic environment between the two sheets of C2B’s β-sandwich motif, the FLT denaturation method should report mostly on the stability of C2B in the C2AB construct [40]. The lack of a discernible transition under these conditions suggests that Ca^2+^ and PS destabilize C2B through negative coupling to such an extent that its β-sandwich tryptophan cannot articulate typical signal change during the unfolding process.

DSC denaturations of C2AB under the analogous PS and Ca^2+^ conditions lend further support to this negative coupling hypothesis. C2B, presumably the first peak in the heat capacity profile of Figure 4H (compare panels Fig. 2D and Fig. 4H), was far less prominent than C2A in the heat capacity profile. Furthermore, the fact that Fig. 4H shows two peaks is, in itself, an illustration of coupling modulation. If strong coupling is present, one peak is seen; if weak coupling is present, two peaks are seen [25]. The C2AB construct unfolds as one coalesced peak under all conditions involving a single ligand, indicating that the energetic parameters describing C2AB combine in a way that facilitates coupling. The combination of PS and Ca^2+^ ligands will, however, modulate the energetic parameters of coupling so that two peaks start to become visible in the denaturation profile.

In contrast to the change in free energy brought about by binding of PS, PIP_2_ had an overall stabilizing effect in the C2AB construct (as indicated by the increase in free energy from 2.24 to 2.56 kcal/mol). There were, however, discrepancies between DSC and FLT denaturation profiles. We attribute these differences to the sensitivity of FLT to microenvironment. Because PIP_2_ is a specific ligand for C2B, its binding might select for a subset of conformers in which more water is excluded from the β-sandwich interior. This in turn might make the tryptophan residue that resides there (Trp 390) more sensitive to changes in solvation in response to increasing temperature and thus account for an early unfolding transition relative to DSC. In the case of PIP_2_ and Ca^2+^ where the DSC profile is somewhat suggestive of independent domain unfolding, not seeing two independent transitions on FLT may be the result of unequal tryptophan signal contribution. Because PIP_2_ is a ligand for C2B, it binding would presumably destabilize C2A through negative coupling, an effect perhaps accentuated by the additional presence of calcium ion [41]. The already superficial tryptophan on C2A (Trp 259) would be even less likely to report on the folded state of C2A.

In this denaturation study, two distinctly different techniques were used to monitor the unfolding transition of each protein construct. The intent of using both DSC (which provides a global perspective) and FLT (a more local perspective) was to overcome any inherent bias of using a single technique in isolation. When comparing the global fit of all DSC and FLT replicates to each individual method, minor deviations of the model were observed in DSC plots of the individual C2 domains. These observations suggest that, indeed, neither technique fully captures the unfolding transition on its own. In the case of C2AB, the unique perspective of each denaturation method is particularly prominent, as noted above. By including both DSC and FLT data sets in the global fit, these unfolding transitions are more completely described. Furthermore, the global fit approach is validated by the close agreement of calculated and calorimetric enthalpies (ΔH_Tm_/ΔH_cal_ ratio in Table 3). For both C2A and C2AB, this ratio was near unity. The higher ΔH_Tm_/ΔH_cal_ ratio of C2B indicates that its unfolding transition has a greater degree of complexity. However, the global two-state model still provides a quantitative estimate of stability and will not change the overall finding of negative coupling and its implications.

When considering the work presented here, it is important to note how the approach used and the results obtained differ from those of previous experimentation looking at domain interaction. Both the free energy of stability and free energy of interaction are global perspectives of Syt I behavior. As such, any specific structural contact points [18], [19] that underlie the apparent malleability of negative coupling are included within Δg_int_. The methodology employed here does not assign defined structural pathways of signal transduction, it encompasses them. In the previous domain interaction studies where no interaction was identified [9], [21], the biophysical methods employed may not have been ideally suited for detecting the small energetic value of Δg_int_. By approaching the question through stability determination as in this study, the small energetic value was more readily apparent because the free energies measured simply did not add up.

With these distinctions in mind, the above observations and thermodynamic profiles can introduce additional insight into Syt I function. The small energetic values of both ΔG°_37°C_ and Δg_int_ suggest sensitivity not only to endogenous ligands, but also structural changes within the protein, like those that arise from gene mutation. Indeed, Syt I’s functional sensitivity to mutation has been well documented [8], [42], [43]. However, point mutations may, as suggested in earlier *in vitro* work [22], negatively impact more than their original intended target. Specifically, point mutations may disrupt inter-domain coupling. This possibility makes assigning adverse physiological affects to what were originally perceived as being localized disruptions difficult because the nature of inter-domain coupling is not local; the free energy terms that facilitate coupling, as they relate to conformational redistribution, are global. A point mutation that interferes with C2B’s membrane binding ability, for instance, would not only disrupt that specific binding event, but also the way in which binding is coupled to modulation of the adjacent C2A domain. Though the C2A domain is not mutated, its function would still be impaired. Recent *in vitro* work, wherein a single C2B point mutation significantly disrupted the otherwise fast synchronous fusion of synthetic fluorescence vesicles in response to injected Ca^2+^, is consistent with this notion [44]. α-synuclein, another allosteric neuronal protein that exhibits a similar form of coupling to C2AB, further exemplifies this type of global sensitivity [34]; oxidation of tyrosine residues far from α-synuclein’s lipid binding domain disrupts the protein’s ability to bind membrane. When elucidating functionality through mutation, these global effects should be considered.

In the context of normal neurotransmission, the large and malleable conformer ensemble of Syt I has further functional implications. If the conformer ensembles of C2A and C2B are subject to modulation by ligands, they may, by extension, be influenced by other domain-specific binding partners in the immediate vicinity within the cell. Indeed, recent EPR and FRET studies looking at Syt I interactions with SNARE proteins show that even when bound to the SNARE complex, structural fluctuation and conformer heterogeneity still exist, indicating yet another possible means for modulating the Syt I conformer ensemble [45], [46]. Experimental evidence on transcriptional protein systems that employ similar models for coupling support this possibility, showing that non-ligand binding partners can exert allosteric influence [36], [37]. Accumulating experimental evidence for the convergence of lipid, Ca^2+^, and fusion machinery proteins on Syt I has long suggested a regulatory role [47]–[49]. By being marginally stable, Syt I may have such capacity, coordinating the molecular events of fusion using the inversely inter-linked, malleable properties of both domains to interact differentially with the wide array of binding partners encountered throughout fusion [50]. This hypothesis, constrained both by the thermodynamic evidence presented here and by extensive experimental data from the synaptotagmin field [6]–[8], [14]–[18], [20], [22], [42]–[46], [51], may provide the necessary integrative means for Syt I-mediated neurotransmitter release.

## Materials and Methods

### Materials

Potassium chloride (KCl) was Puriss-grade. Calcium chloride dihydrate, 3-(N-morpholino) propanesulfonic acid (MOPS), and ethylene glycol-bis(2-aminoethyl)-N,N,N′,N′tetra-acetic acid (EGTA) were all Biochemika grade from Fluka Chemical Corp. All buffers were decalcified using Chelex-100 ion-exchange resin (Bio-Rad Labs). All lipids including 1-palmitoyl-2-oleoyl-*sn*-glycero-3-phosphocholine (POPC, 16∶0,18∶1PC), 1-palmitoyl-2-oleoyl-*sn*-glycero-3-phosphoserine (POPS, 16∶0,18∶1PS), and 1-stearoyl-2-arachidonoyl-*sn*-glycero-3-phospho-(1′-myo-inositol-4′,5′-bisphosphate) (PIP_2_, 18∶0,20∶4PI(4,5)P_2_) were obtained from Avanti Polar Lipids (Birmingham, AL).

### Preparation of Lipid Vesicles

Large unilamellar vesicles (LUVs) consisting of POPC:POPS (60∶40) and POPC:PIP_2_ (95∶5) were prepared as previously described [26]. PIP_2_-containing vesicles were not, however, lyophilized. Concentrations for all lipid stock solutions were verified using a phosphate assay [52].

### Protein Purification

Human Syt I C2A and C2AB constructs were purified according to previously described methods [26], [53]. Human Syt I C2B was expressed and purified as a maltose-binding fusion protein. Full details of this domain’s purification can be found in Text S1 (Fig. S2). Final protein concentrations prior to use in denaturation scans were determined using a Nanodrop (ThermoScientific) with each construct’s respective A280 extinction coefficient.

### DSC

DSC experiments were performed on a NanoDSC (TA Instruments, New Castle, DE) at a scan rate of 1°C/min. To see if measured enthalpies varied with concentration or scan rate, all constructs were denatured over a range of concentrations and scan rates [26]. In C2A + C2B DSC control scans, concentrations of each domain were equimolar. All scans were conducted in chelexed 20 mM MOPS, 100 mM KCl, pH 7.5. Scans performed in the absence of Ca^2+^ contained 500 µM EGTA. Scans of both C2A and C2B domains in the presence of Ca^2+^ had ligand concentrations sufficient for >95% saturation of Ca^2+^-binding sites (binding constants obtained from both binding studies [54] with terbium ion and from isothermal titration calorimetry studies). For the C2AB construct, a Ca^2+^ concentration of 5.2 mM was used. The concentration of Ca^2+^ stock solution used for all scans was verified using both a calcium ion selective electrode (ThermoScientific) and a BAPTA chelating assay (Invitrogen/Molecular Probes, Eugene, OR). Scans carried out with lipid contained LUVs composed of either a 60∶40 mixture of POPC:POPS or a 95∶5 mixture of POPC:PIP_2_. Excess ligand was intentionally used to thermodynamically characterize a specific subset of conformers (only conformers that bind Ca^2+^, for instance) and not a heterogeneous population of conformations (a mixture of ligand-bound and unbound Syt I). To apply reversible thermodynamics, some evidence of reversible protein folding is needed. By comparing the measured enthalpies of the first and second scans of a single DSC sample, a percent reversibility was determined.

### FLT

FLT experiments were performed on a Lifetime Spectrometer (Fluorescence Innovations, Bozeman, MT) using nanomolar protein concentrations. Under some ligand conditions, higher concentrations were needed to obtain good signal. Scans were conducted in chelexed 20 mM MOPS, 100 mM KCl, pH 7.5. No time-resolved measurements were made. Instead, the integrated intensity of the lifetime decay was used to selectively monitor intrinsic fluorescence of endogenous tryptophan residues (excitation and emission wavelengths of 295 and 340 nm, respectively). Change in fluorescence emission for each construct was monitored as a function of increasing 2°C temperature increments. In scans free of Ca^2+^, 500 µM EGTA was added as background. In scans with Ca^2+^, both C2A and C2B binding sites were >95% saturated (as described above in DSC section). For C2AB, a Ca^2+^ concentration of 5.1 mM was used. The same Ca^2+^ stock solution described above was used for FLT samples. Scans with lipid contained LUVs of identical composition as described above for DSC. Percent reversibility was measured by comparing the integrated fluorescence lifetime intensity of the sample before heating and after cooling. All data sets were analyzed at an emission wavelength of 345 nm to verify absence of contributing water fluorescence at 340 nm. The fluorescence signal measured was normalized to the calculated intensities from the two-state model and subsequently displayed as “Normalized Intensity” in each corresponding plot.

### Global DSC and FLT Analysis

The free energy of stability for each construct under each set of ligand conditions was determined by globally fitting denaturation data from DSC and FLT methods to a two-state transition model. For full details of this model and its application, see Text S1. In this model, there is equilibrium between native (N) and denatured (D) states, represented by the equilibrium constant below:
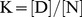
(1)


The equilibrium constant can be used to represent fractions of folded (f_N_) and unfolded (f_D_) protein throughout the transition. As a protein undergoes thermal denaturation in the DSC, the heat capacity (C_p_(T)) of the sample cell changes as the fraction of unfolded protein changes:
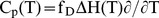
(2)


Where ΔH(T) is the associated enthalpy. When the protein is denatured in FLT, the tryptophan residues become more solvent exposed and lose much of their initial intensity. Throughout the unfolding transition, the total fluorescence signal measured (S(T)) is a composite of native (S_N_) and denatured (S_D_) protein fluorescence and depends on the fraction of each present at a given temperature:
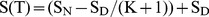
(3)


Where S_N_ and S_D_ are approximated by using linear equations. By substituting the Gibbs-Helmholtz equation (equation (4)) into equation (3) and equation (2) above and making simple rearrangements, data from both methods were fit simultaneously using a non-linear least squares regression approach (see Text S1) [55].
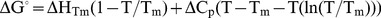
(4)


The terms ΔH_Tm_, T_m_, and ΔC_p_ are the fit parameters in this model, however, to further constrain this fit, ΔC_p_ was fixed using an empirical approximation method that showed good agreement with experimentally-derived values [56]. The fixed ΔC_p_ values for C2A, C2B, and C2AB were 1.92, 2.19, and 4.53 kcal/mole, respectively. The parameters resulting from the global fit were subsequently substituted into equation (4) to determine the free energy of stability at any specified temperature (T) which in this study was the physiological temperature of 37°C. Four replicates of each technique (for a total of *n = *8, when achievable) were used in the global fit for each set of conditions. Each data point within each technique is the average of the 4 replicates with the associated error plotted as 95% confidence interval. The error associated with fit parameters and the model line, which represents the global fit for all 8 data sets, were determined using the SolverAid function of Excel in an approach by De Levie [57].

Lastly, the entropy change associated with the unfolding transition (ΔS) was calculated using the ΔG°_37°C_, ΔH_Tm_, and the physiological temperature of 310 K of each protein construct under each set of ligand conditions using the Gibbs free energy equation:
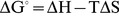
(5)


## Supporting Information

Figure S1 Application of the fluctuation dissipation theorem to C2AB. Qualitative representation of native (N) and denatured (D) enthalpy distributions for C2AB in the absence of any ligand (blue), in the presence of POPC:POPS (60∶40) liposomes (green), and in the presence of Ca^2+^ (red). Note that the enthalpy distributions of an N/D pair shift in response to ligand, consistent with C2AB malleability and a ligand-induced change in conformer distribution.
(TIF)

Figure S2 Effectiveness of the C2B purification protocol used for this study. Prominent band in lanes 2, 3, and 5 are MBP-C2B after cell lysis (2), during elution of unwanted protein (3), and during 250 mM imidazole elution (5). Lane 6 shows separation of MBP from C2B. Lane 7 shows pure C2B after passing cut MBP-C2B over column. Ladder (lanes 1 and 8, Precision Plus, Bio-Rad Labs) has molecular weights (kDa) of 10, 15, 20, 25, 37, 50, 75, 100, 150, and 250.
(TIF)

Table S1 Complete list of calorimetric enthalpies used to assess concentration dependence of the C2B domain.
(DOC)

Table S2 Complete list of calorimetric enthalpies used to assess concentration dependence of the C2AB cytosolic fragment.
(DOC)

Table S3 Thermodynamic parameters and associated errors for C2B and C2AB using FLT λ_em_ of 345 nm. Analogous controls were performed for C2A. When FLT integrated fluorescence intensity was normalized and globally fit, there was no substantial variation in calculated fit parameters. This indicated that water fluorescence (water raman at 328 nm) at 340 nm was negligible.
(DOC)

Text S1 Two-state derivation, heat capacity fluctuation dissipation theorem, C2B purification, and non-linear least squares regression analysis.
(DOC)
