# Supplementary figures and images for: Negative Coupling as a Mechanism for Signal Propagation between C2 Domains of Synaptotagmin I
Source: PLoS One. 2012 Oct 5;7(10):e46748. doi: 10.1371/journal.pone.0046748 (PMC3465270; doi:10.1371/journal.pone.0046748)

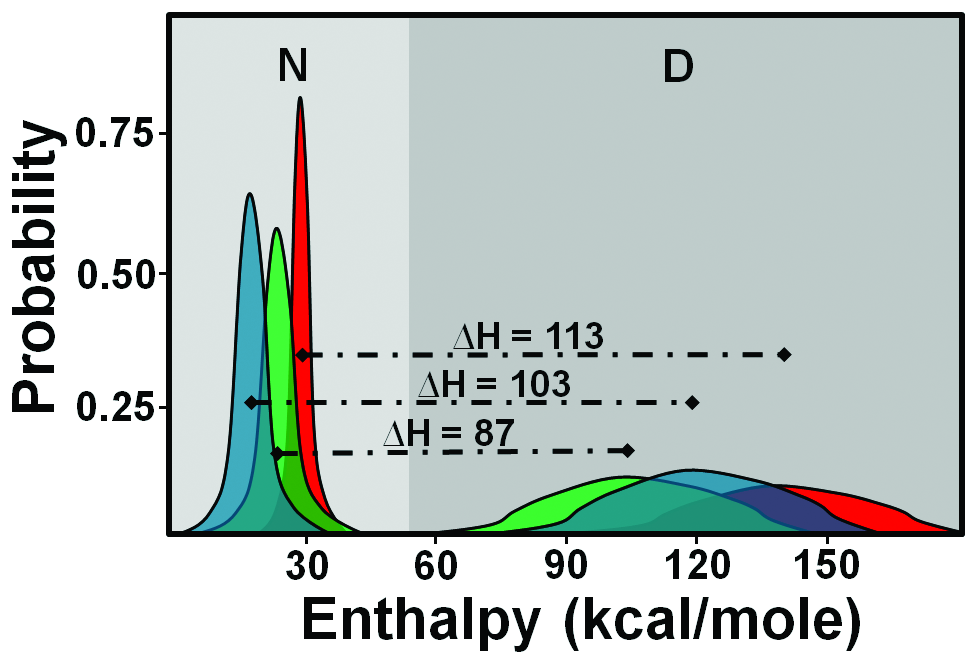

Supplement: Figure S1 — Application of the fluctuation dissipation theorem to C2AB. Qualitative representation of native (N) and denatured (D) enthalpy distributions for C2AB in the absence of any ligand (blue), in the presence of POPC:POPS (60∶40) liposomes (green), and in the presence of Ca2+ (red). Note that the enthalpy distributions of an N/D pair shift in response to ligand, consistent with C2AB malleability and a ligand-induced change in conformer distribution. (TIF) [file pone.0046748.s001.tif]

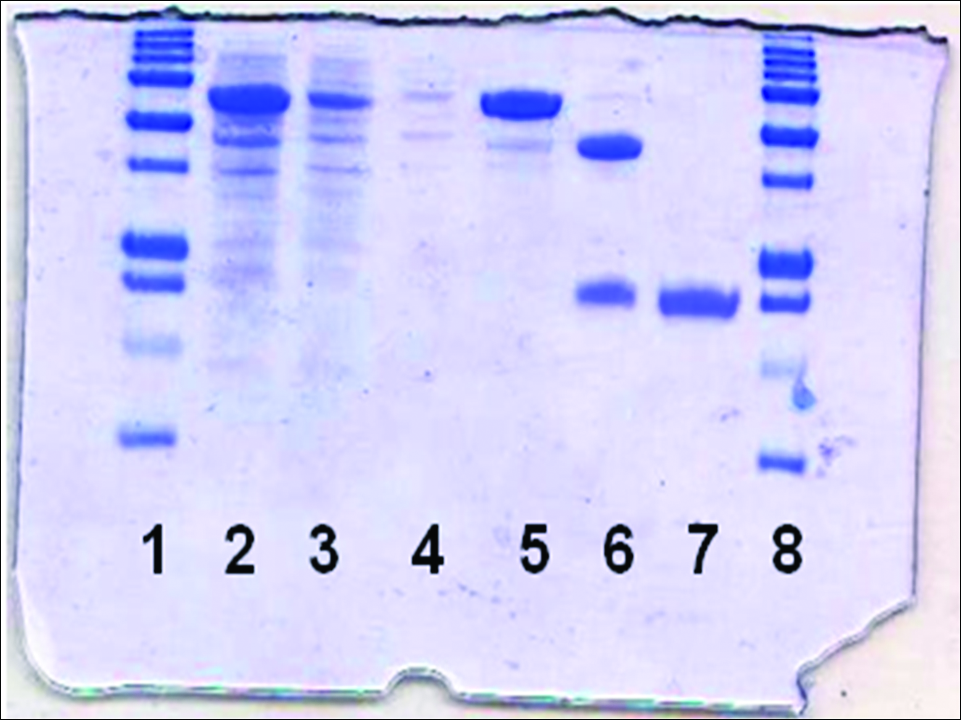

Supplement: Figure S2 — Effectiveness of the C2B purification protocol used for this study. Prominent band in lanes 2, 3, and 5 are MBP-C2B after cell lysis (2), during elution of unwanted protein (3), and during 250 mM imidazole elution (5). Lane 6 shows separation of MBP from C2B. Lane 7 shows pure C2B after passing cut MBP-C2B over column. Ladder (lanes 1 and 8, Precision Plus, Bio-Rad Labs) has molecular weights (kDa) of 10, 15, 20, 25, 37, 50, 75, 100, 150, and 250. (TIF) [file pone.0046748.s002.tif]
