# Supplementary material for: Negative Coupling as a Mechanism for Signal Propagation between C2 Domains of Synaptotagmin I
Source: PLoS One. 2012 Oct 5;7(10):e46748. doi: 10.1371/journal.pone.0046748 (PMC3465270; doi:10.1371/journal.pone.0046748)
Supplement: Table S1 — Complete list of calorimetric enthalpies used to assess concentration dependence of the C2B domain. (DOC) [file pone.0046748.s003.doc]

| **Human Syt I C2B** | |
| --- | --- |
| *Concentration (mM)* | *ΔH_cal_ (kcal/mole)* |
| 0.013 | 45.9 |
| 0.014 | 37.5 |
| 0.012 | 53.2 |
| 0.013 | 47.3 |
| 0.014 | 43.0 |
| 0.013 | 48.7 |
| 0.020 | 42.1 |
| 0.015 | 45.7 |
| 0.021 | 43.7 |
| 0.013 | 45.2 |
| 0.012 | 46.9 |
| *Average ΔH_cal_* | 45.4 |
| *Standard Deviation* | 4.0 |

**Table S1.** Complete list of calorimetric enthalpies used to assess concentration dependence of the C2B domain.
