# Supplementary material for: Negative Coupling as a Mechanism for Signal Propagation between C2 Domains of Synaptotagmin I
Source: PLoS One. 2012 Oct 5;7(10):e46748. doi: 10.1371/journal.pone.0046748 (PMC3465270; doi:10.1371/journal.pone.0046748)
Supplement: Table S2 — Complete list of calorimetric enthalpies used to assess concentration dependence of the C2AB cytosolic fragment. (DOC) [file pone.0046748.s004.doc]

| **Human Syt I C2AB** | |
| --- | --- |
| *Concentration (mM)* | *ΔH_cal_ (kcal/mole)* |
| 0.011 | 104.7 |
| 0.013 | 96.1 |
| 0.013 | 94.9 |
| 0.013 | 85.2 |
| 0.013 | 99.6 |
| 0.013 | 85.2 |
| 0.010 | 86.7 |
| 0.012 | 87.7 |
| 0.013 | 93.6 |
| 0.011 | 100.4 |
| 0.015 | 89.0 |
| 0.019 | 85.9 |
| 0.020 | 85.6 |
| 0.011 | 86.2 |
| 0.015 | 90.8 |
| 0.019 | 91.8 |
| 0.020 | 93.4 |
| *Average ΔH_cal_* | 91.6 |
| *Standard Deviation* | 6.0 |

**Table S2.** Complete list of calorimetric enthalpies used to assess concentration dependence of the C2AB cytosolic fragment.
