# Supplementary material for: Negative Coupling as a Mechanism for Signal Propagation between C2 Domains of Synaptotagmin I
Source: PLoS One. 2012 Oct 5;7(10):e46748. doi: 10.1371/journal.pone.0046748 (PMC3465270; doi:10.1371/journal.pone.0046748)
Supplement: Table S3 — Thermodynamic parameters and associated errors for C2B and C2AB using FLT λem of 345 nm. Analogous controls were performed for C2A. When FLT integrated fluorescence intensity was normalized and globally fit, there was no substantial variation in calculated fit parameters. This indicated that water fluorescence (water raman at 328 nm) at 340 nm was negligible. (DOC) [file pone.0046748.s005.doc]

| **Protein (Environment)** | **ΔH_Tm_ (kcal/mole)** | **ΔG° at 37 °C (kcal/mole)** | **ΔS (kcal/mole·K)** | **T_m_ (°C)** | **ΔH_Tm_ /ΔH_cal_** |
| --- | --- | --- | --- | --- | --- |
| *C2B (EGTA)* | 69.2±0.6 | 1.73±0.08 | 0.22±0.01 | 46.4±0.1 | 1.44 |
| *C2B (Ca^2+^)* | 81.9±0.5 | 3.79±0.09 | 0.25±0.01 | 59.0±0.1 | 1.39 |
| *C2B (PS)* | 63.4±0.2 | 1.16±0.01 | 0.20±0.01 | 43.5±0.1 | 1.61 |
| *C2AB (EGTA)* | 103.1±0.4 | 2.25±0.01 | 0.32±0.01 | 45.6±0.1 | 1.06 |
| *C2AB (Ca^2+^)* | 112.8±0.5 | 4.09±0.01 | 0.34±0.01 | 59.2±0.1 | 0.97 |
| *C2AB (PS)* | 86.2±0.3 | 1.68±0.01 | 0.27±0.01 | 44.8±0.1 | 1.05 |
| *C2AB (PIP_2_)** | 188±3 | 4.00±0.60 | 0.59±0.02 | 44.4±0.1 | NA |
| *C2AB (Ca^2+^, PIP_2_)** | 140±1 | 5.80±0.04 | 0.42±0.01 | 58.0±0.1 | NA |

**Table S3.** Thermodynamic parameters and associated errors for C2B and C2AB using FLT λ_em_ of 345 nm. Analogous controls were performed for C2A. When FLT integrated fluorescence intensity was normalized and globally fit, there was no substantial variation in calculated fit parameters. This indicated water fluorescence (water raman at 328 nm) at 340 nm was negligible.
