# Supplementary material for: Negative Coupling as a Mechanism for Signal Propagation between C2 Domains of Synaptotagmin I
Source: PLoS One. 2012 Oct 5;7(10):e46748. doi: 10.1371/journal.pone.0046748 (PMC3465270; doi:10.1371/journal.pone.0046748)
Supplement: Text S1 — Two-state derivation, heat capacity fluctuation dissipation theorem, C2B purification, and non-linear least squares regression analysis. (DOC) [file pone.0046748.s006.doc]

**Supporting Material – Text S1**

**Two-State Model of Protein Unfolding**

In the two-state model of protein unfolding, a conformationally flexible protein exists in either a native (N) or denatured (D) ensemble, wherein conformers of each ensemble have similar energies. These two ensemble states are represented by an equilibrium constant:

K = [D]/[N] (S1)

The equilibrium constant is directly related to free energy of stability (ΔG°, Eq. S2 and Eq. S3). The equilibrium constant is not easily measured directly in the laboratory. Instead, free energy is approached from a calorimetric direction. Free energy has enthalpic (ΔH(T)) and entropic (ΔS(T)) contributions, both of which are temperature dependent functions:

ΔG° = -RTln(K) (S2)

ΔG° = ΔH(T) - TΔS(T) (S3)

ΔH(T) = ΔH_Tm_ +
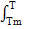
ΔC_p_(T) *dT* (S4)

ΔS(T) = ΔH_Tm_/T_m_ +
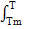
ΔC_p_(T) *dln(T)* (S5)

Where ΔH_Tm_ is a reference enthalpy typically obtained from a calorimeter, T_m_ is a reference temperature (typically the melting temperature of the protein), and ΔC_p_(T) is the difference in sample cell heat capacity between the native and denatured ensemble states. If ΔC_p_(T) is assumed to be temperature independent (designated by the notation ΔC_p_), Eq. S4 and Eq. S5 can be substituted into Eq. S3 to obtain the Gibbs-Helmholtz equation:

ΔG° = ΔH_Tm_(1 – T/T_m_) + ΔC_p_[T – T_m_ – T(ln(T/T_m_))] (S6)

Eq. S6 enables calculation of protein stability at any temperature (T).

At any point in the unfolding transition, the fraction of native (f_N_) and denatured (f_D_) protein can be determined using mass balance for total protein (P_T_) and the equilibrium constant:

P_T_ = [D] + [N] (S7)

f_N_ = [N]/P_T_ = [N]/([D] + [N]) = 1/(K + 1) (S8)

f_D_ = [D]/P_T_ = [D]/([D] + [N]) = K/(K + 1) (S9)

As a protein thermally denatures in a differential scanning calorimeter (DSC), the heat capacity (C_p_(T)) of the sample cell changes as the fraction of unfolded protein changes:

C_p_(T) = f_D_ ΔH(T) *d/dT* (S10)

Application of the Product Rule expands the above derivative into two terms:

C_p_(T) = (f_D_(*d/dT*))ΔH(T) + f_D_(ΔH(T)(*d/dT*)) (S11)

Differentiating Eq. S4, assuming ΔC_p_(T) is a temperature-independent function, simplifies the expression to ΔC_p_. Differentiating f_D_ requires further use of the Quotient Rule and substitution of nested temperature-dependent functions (Eq. S2 and Eq. S6 into Eq. S9):

f_D_ (*d/dT*) = (*e*^-(ΔHTm(1 – T/Tm) + ΔCp[T – Tm – T(ln(T/Tm))])^/

*e*^-(ΔHTm(1 – T/Tm) + ΔCp[T – Tm – T(ln(T/Tm))])^ + 1) *d/dT* (S12)

f_D_ (*d/dT*) = [((ΔH_Tm_/T – ΔC_p_T_m_/T + ΔC_p_)/RT)((K(K + 1)) - (K^2^/RT))]/

((K+1)^2^) (S13)

Substituting Eq. S13 back into Eq. S11 generates the two-state model used to fit DSC data:

C_p_(T) = ΔH(T)([((ΔH_Tm_/T – ΔC_p_T_m_/T + ΔC_p_)/RT)((K(K + 1)) - (K^2^/RT))]/ ((K+1)^2^)) + (ΔC_p_K/(K + 1)) + FBL_DSC_ (S14)

Where FBL_DSC_ refers to the folded baseline heat capacity determined with a linear equation.

To obtain a more stringent and complete fit, denaturation data from FLT was simultaneously fit to the same model. FLT selectively monitors intrinsic fluorescence of tryptophan residues (Trp) in a protein. In the native state, the environment surrounding Trp allows for maximum fluorescence. When the protein denatures, the Trp become solvent exposed and lose much of their initial intensity. Throughout the transition, the total signal measured (S(T)) is a composite of native (S_N_) and denatured (S_D_) protein fluorescence:

S(T) = S_N_ + S_D_ (S15)

The contribution from both native and denatured states depends on the fraction of each present at a given temperature. The total integrated fluorescence lifetime signal can be expressed as such:

S(T) = S_N_/(K + 1) + S_D_ (S16)

S_N_ and S_D_ are determined with linear functions before (the folded baseline, FBL_FLT_) and after (the unfolded baseline, UBL) the unfolding transition:

S_N_ = FBL_FLT_ = (m_N_)T + int_N_ (S17)

S_D_ = UBL = (m_D_)T + int_D_ (S18)

Where m and int are the slope and intercept, respectively, for each baseline. To determine S_N_ throughout the course of the denaturation, the signal contributed by S_D_ must be subtracted off the measured composite signal. The model for FLT signal as a function of temperature becomes:

S(T) = (FBL_FLT_ – UBL)/(K + 1) + (UBL) (S19)

By substituting a rearranged Eq. S2 and Eq. S6 into Eq. S19, the fitting of FLT and DSC denaturation data are linked and can be subject to non-linear least squares regression.

**Fluctuation Dissipation Theorem**

The fluctuation dissipation theorem describes fluctuations about a mean for a given system. In the case of protein denaturation where heat capacity (C_p_) is measured, the enthalpy fluctuates and the theorem is [1]:

C_p_ = (H^2^ - <H>^2^)/RT^2^ (S20)

Where H is enthalpy in a Gaussian distribution about the mean <H>, R is the gas constant, and T is temperature. C_p_ is thus proportional to the enthalpy deviation (H^2^ - <H>^2^) of either folded or unfolded states of a protein. The heat capacities for native and denatured states differ due to the extent of hydrophobic residue exposure in aqueous solution. As such, the native H^2^ - <H>^2^ is much smaller than the denatured H^2^ - <H>^2^. Using the folded and unfolded heat capacity baselines obtained from the DSC, the relative widths of the folded and unfolded enthalpy distributions can be approximated.

The distance between the mean enthalpies of each distribution is directly measured by the calorimeter and is the change in enthalpy (ΔH) between folded and unfolded states of the protein. By combining estimates for folded C_p_ and unfolded C_p_ with the change in enthalpy, enthalpy distribution plots can be sketched (Fig. S1). The assembly of these plots allows for qualitative monitoring of ligand-induced changes in the protein’s native and denatured enthalpy distributions.

**Purification of Isolated C2B Domain**

The C2B purification was similar to C2A and C2AB with respect to plasmid preparation, *E. coli* transformation and growth, cell lysis, and nucleic acid degradation. However, C2B was expressed with a 6xHis-tagged maltose-binding protein (MBP). The amino acids linking C2B to MBP formed a cleavage site for a 6xHis-tagged tobacco etch virus (TEV) protease. After overnight treatment with benzonuclease at 4 °C, C2B-MBP fusion protein was equilibrated with slurried Ni-NTA agarose media (Qiagen) in 20 mM MOPS/100 mM KCl/10 mM imidazole/pH 7.5 buffer for 3 hours at 4 °C. The 10 mM imidazole prevented non-specific protein binding. After equilibration, the slurried media was transferred into a column. Complete elution of undesired protein was monitored by measuring the A280 using a Nanodrop (Thermo Scientific). To remove nucleic acid, a 10 column volume wash was carried out with 20 mM MOPS/1M KCl/pH 7.5 buffer. The column was re-equilibrated with the 10 mM imidazole buffer. The nucleic acid-free fusion protein (A260/A280<0.8) was then eluted with 20 mM MOPS/100 mM KCl/250 mM imidazole/pH 7.5 buffer. Eluted MBP-C2B was dialyzed with TEV protease in 20 mM MOPS/100 mM KCl/pH of 7.5 buffer (3 mg of TEV protease were added for every 150 mg of fusion protein). After >14 hours, cut MBP-C2B and TEV protease were passed over the column. C2B was collected in pass through and its purity was assessed with the Nanodrop (A260/A280<0.8) and gel electrophoresis (Fig. S2). Pure C2B was dialyzed in chelexed Fluka grade 20 mM MOPS/100 mM KCl/pH 7.5 buffer prior to use in denaturation studies. Concentrations were determined using an A280 extinction coefficient of 19060 cm^-1^ M^-1^.

**Non-Linear Least Squares Regression Analysis**

The non-linear least squares regression analysis is a fitting method that matches the output of Eq. S14 and Eq. S19 to the experimental data by manipulating fit parameters (ΔH_Tm_, ΔC_p_, T_m_, m_N_, int_N_, m_D_, int_D_) within the model equations. The close fit is accomplished in Excel through use of the Solver, which minimizes the total sum of the square differences (Σ(experimental signal – model signal)^2^) to fit the data. Error associated with the resulting fit can then be determined with the macro-functions SolverAid, which estimates error using 1) the scaling factor by with the fit parameters were changed, 2) the global sum of the square difference (for all 8 data sets) after fitting has occurred, and 3) the model’s numerical outputs for signal (the theoretical heat capacity or fluorescence signal). For more detail on analysis, see: [2,3].

**Supporting References**

1. Hill TL (1986) An introduction to statistical thermodynamics. Toronto: General Publishing Company, Ltd.

2. De Levie R (1999) Estimating parameter precision in nonlinear least squares with excel's solver. J Chem Educ 76: 1594-1598.

3. Streicher WW, Makhatadze GI (2007) Unfolding thermodynamics of Trp-cage, a 20 residue miniprotein, studied by differential scanning calorimetry and circular dichroism spectroscopy. Biochemistry 46: 2876-2880.
